# Color Choice Preference in Cognitively Impaired Patients: A Look Inside Alzheimer’s Disease Through the Use of Lüscher Color Diagnostic

**DOI:** 10.3389/fpsyg.2019.01951

**Published:** 2019-08-27

**Authors:** Michelangelo Stanzani Maserati, Micaela Mitolo, Federica Medici, Renato D’Onofrio, Federico Oppi, Roberto Poda, Maddalena De Matteis, Caterina Tonon, Raffaele Lodi, Rocco Liguori, Sabina Capellari

**Affiliations:** ^1^IRCCS Istituto delle Scienze Neurologiche di Bologna, Bologna, Italy; ^2^Dipartimento di Scienze Biomediche e NeuroMotorie, Università di Bologna, Bologna, Italy

**Keywords:** Alzheimer’s disease, dementia, mild cognitive impairment, color preference, Lüscher color test, personality

## Abstract

**Objective:**

To study the emotional state of cognitively impaired patients through the color choice preference in a group of Alzheimer’s disease (AD) patients and compare it with a group of Mild Cognitive Impairment (MCI) patients and a matched control group.

**Methods:**

A total of 71 AD, 50 MCI and 68 controls were consecutively evaluated. All patients and controls underwent the Mini Mental State Evaluation (MMSE) and the Lüscher color test.

**Results:**

Cognitively impaired patients mainly chose auxiliary colors, in particular violet and brown, and rejected black and gray. AD patients predominantly chose forms corresponding to auxiliary colors. The auxiliary color choice negatively correlated with the MMSE score. MCI patients and controls had a higher presence of anxiety on gray table and controls had higher frustration and ambivalence, i.e., psychic complexity, on basic color tables.Data globally suggest that AD patients live with a feeling of personal change due to instability and emotional insecurity, experiencing physical discomfort and a bodily need of being welcomed in a favorable environment. They aspire to a sensitive understanding by someone with whom they can be identified. Differently, MCI patients have less of these needs; however, they feel more anxious.

**Conclusion:**

The comprehension of the inner emotional state of cognitively impaired patients allows us to better communicate with them and effectively approach their behavioral disorders. Like other projective techniques, such as the tree-drawing test and the human figure-drawing test, Lüscher color test is proposed as a simple and unconventional approach to understand the emotional life of AD patients. The awareness of clinicians about the existential fragility and insecurity of such type of patients allows us not only to better manage their behavioral disturbances but also to improve their quality of life and that of their caregivers.

## Introduction

Alzheimer’s disease (AD) is a neurodegenerative disorder, primarily affecting memory and attention, that leads to a progressive global cognitive dysfunction. It is the main cause of dementia accounting for 50–75% and doubling in prevalence every 5 years after age 65 (Lane et al., 2018). AD is usually preceded by mild cognitive impairment (MCI), a clinical prodromal syndrome that is an intermediate stage between the expected cognitive decline of normal aging and the very earliest features of dementia (Albert et al., 2011), representing a useful condition to compare to AD since its risk of evolution into dementia. The rate of conversion from MCI to dementia is heterogeneous. In our Cognitive Disorders Center the rate of conversion is 18.4% in a 4-year follow-up (Gallassi et al., 2010) while in literature higher annual rates of progression are reported (6–15%) (Petersen et al., 2009). Thus, MCI and AD represent a continuum from the mildest degree of cognitive impairment to dementia.

AD affects not only cognitive functions but also emotional processing leading to behavioral dysregulation and progressive deficits in social functioning disrupting daily life activities and causing social isolation (Fischer et al., 2019). Thus, interpersonal behavioral changes are likely to be the main cause of interpersonal stress affecting both the patient and the caregiver (Clare et al., 2012). Nevertheless, if compared with other dementias, there is evidence of some preservation of emotional competencies and interpersonal functioning in AD patients (Fernandez-Duque et al., 2010; Dermody et al., 2016).

Exploring the emotional state of such type of patients is therefore important to learn more about their feelings and communicate with them properly, fulfilling their wishes and minimizing pharmacological treatment. Since verbal communication can be difficult over the course of AD due to language deficits that interfere with verbal expression and comprehension, simple tools exploring the inner emotional state that require minimal cognitive involvement, especially of language, are thus desirable.

In this perspective, personality and affectivity, as the background of every behavior, can be studied in cognitively impaired patients in order to point out different types and degrees of cognitive impairment (Stanzani Maserati et al., 2015, 2018). Thus, studying personality and affectivity in such type of patients can be a useful access to infer their needs and subjective emotional states.

Projective techniques for the study of personality and affectivity are instruments of simple administration with the potential to involve unconscious psychic dynamics both in the pediatric and adult life. Among these tools, Lüscher color test is used for exploring the emotional state through an unconscious selection of color preferences (Lüscher and Scott, 1969). Considering that it is a non-verbal tool and that it does not require specific cognitive skills to be performed, it could be a rapid and simple way to access the emotional states of cognitively impaired patients.

Firstly described by Lüscher (1948), the test is a projective technique widely used for orienting psychodiagnosis. Despite its diffusion in clinical practice, its validity has been widely discussed showing its limitations (Donnelly, 1974, 1977; Braün and Bonta, 1979; Stimpson and Stimpson, 1979; Corotto and Hafner, 1980; Holmes et al., 1984, 1986; Picco and Dzindolet, 1994; Kertzman et al., 2003). Because of this critical issue, Lüscher test is more suitable to explore the emotional state of a patient rather than its personality structure and this is the limit through which it was used in this work.

At its foundation there is the assumption that colors are objective stimuli whose psychological meanings are universal but each individual relates to color depending on their psychovegetative and existential state. Faced with the objective color stimuli of the test, subjects react in a personal way by determining the variables that, transcribed in the protocol, allow an adequate interpretation (Lüscher and Scott, 1969). Lüscher color test involves the administration of a series of choices of colored squares distributed in a series of tables selected by the author on an empirical basis. Colors can be preferred or rejected depending on the order of choice. Finally, the resulting order of color choices is matched to descriptive statements that supposedly give a description of the affectivity of the subject examined (Lüscher and Scott, 1969). Therefore, in order to test differences of emotional states in selected groups of cognitively impaired patients, we evaluated color choice preferences in a group of AD patients and compared the results with those of a group of MCI patients and a group of controls.

## Materials and Methods

We evaluated consecutive outpatients, 71 AD and 50 MCI, referred for cognitive disorders over a 1-year period by their relatives and physicians or who spontaneously presented themselves to the Cognitive Disorders Center of IRCCS Istituto delle Scienze Neurologiche of Bologna, Italy. Patients were compared with a group of 68 controls matched for age, sex, and education.

### Inclusion and Exclusion Criteria

Patient inclusion criteria were as follows: (a) major or minor neurocognitive disorder according to DSM-V criteria (American Psychiatric Association, 2013); (b) diagnosis of AD and MCI based on the international criteria (Petersen et al., 2009; McKhann et al., 2011).

Patient exclusion criteria were: (a) current or previous neurological, psychiatric and systemic diseases; (b) alcoholism or other substance abuse; (c) history of color blindness or a history of diseases with a significant impact on color vision (e.g., severe glaucoma, progressive cone dystrophy, severe cataract or macular degeneration); (d) use of neuroleptics or other antipsychotics and tricyclic antidepressants considering their possible negative effects on cognition; (e) evidence of visual agnosia, hemispatial neglect, relevant apraxia or relevant verbal comprehension deficits on neurological examination.

A group of controls, matched for age, sex, and education, was selected. These subjects did not have any past or present neurological, psychiatric or general diseases, alcoholism or other substance abuse, history of color blindness or diseases with a significant impact on color vision. They were selected mainly from the relatives of the patients. Caregivers of patients were excluded considering a possible interference of anxiety and depression.

All patients and controls gave their informed consent to the study according to the Declaration of Helsinki.

### Procedures

All patients and controls underwent a general cognitive screening with the Mini Mental State Examination (MMSE) (Folstein et al., 1975) and a full version of the Lüscher color test (Lüscher and Scott, 1969) (©Copyright 1949 and 2008 by Color-Test-Verlag AG, Theaterstrasse 1, CH-6003 Luzern). The MMSE was administered to patients and controls by an examiner blind to the patient’s diagnosis and Lüscher color test results. The MMSE score was corrected for age and education according to Italian standardizations (Magni et al., 1996).

### Lüscher Color Test

Lüscher color test consists in the administration of a series of choices of colored squares distributed in a series of eight tables plus a choice of simple forms in a separate table (Lüscher and Scott, 1969). No limits of time are given. Colors can be preferred or rejected depending on the order of choice. Eight colors are selected: four basic colors (blue, green, red and yellow) that underlie basic physiological and psychological needs such as calm, stability, motivation, availability and four auxiliary colors (violet, brown, black and gray) that show integrative characteristics of the personality like sensitiveness, relaxation, coercion and numbness (Table 1).

**TABLE 1:** Lüscher color test’s eight colors, the associated forms, and their meanings.

**Colors**		**Associated form**	**Physiological meaning**	**Psychological meaning**
Basic	1. Blue	A black circle on a white background	*Quietude*	*Membership*
	2. Green	A little dark square surrounded by a bigger one	*Constraint*	*Steadiness*
	3. Red	A dark-contour acute triangle	*Excitement*	*Activity*
	4. Yellow	A white circle on a dark background	*Relief*	*Openness*
Auxiliary	5. Violet	A rhombus with a dark contour and rotund sides	*Sensitiveness*	*Transformation*
	6. Brown	A sine curve on a dark background	*Relaxation*	*Well-being*
	7. Black	−	*Stasis*	*Coercion*
	0. Gray	A dark hexagon on a white background	*Numbness*	*Spacing*

*From The Lüscher Color Test, modified (Lüscher and Scott, 1969).*

The choice of colors in the test is made explicitly in four tables where the squares are colored directly with the eight colors or implicitly in other four specific tables where the squares are colored with a basic color shaded with one of the other basic colors. Globally, when the subject can choose all the colors in order of preference in the same table, basic colors usually appear in the first four selections while auxiliary colors are usually expected to fall within the last four selections. Only one table provides the choice of simple black and white shapes that correspond to basic and auxiliary colors (Table 1). Shapes represent the behavior that the subject implements in the context expressed by the color. If there is a contradiction between the choice of a favorite color and the corresponding rejected form or vice versa, then there is a behavioral mask that is a contrast between the inner emotional state and the behavior that the subject adopts to deal with it.

Specifically, the test is composed of four parts: **Gray table:** consists of five squares, three of different shades of gray, one black and one white. Subjects are asked to choose in order of preference three different squares: two preferred and one rejected. If the choice of each square fits with an expected result, this is an indicator of the anxiety in dealing with the requested task (i.e., state anxiety). **Eight color table:** consists of eight squares of different colors, four basic (blue, green, red, yellow) and four auxiliary (violet, brown, gray, black). Subjects are asked to choose in order of preference all eight colors. This choice is requested twice: the first time after the gray table, the second at the end of the test. The second choice is considered more reliable (Lüscher and Scott, 1969). The choice of the preferred color in the first position and the one rejected in the last are respectively considered in the second choice as an indicator of desired objectives and suppressed characteristics. The rejection of a basic color in the second choice is considered an indicator of the anxiety that the patient experiences in everyday life (i.e., trait anxiety). At the end of the second choice subjects are also asked to indicate the “combined color” that is the color that best fits with the favorite color and symbolizes the desire that the subject wants to achieve. **Form table:** consists of seven different black and white forms each one corresponding to a basic or an auxiliary color, except black. Subjects are asked to choose forms indicating which two they like most and which two they dislike most. Behavioral masks (i.e., contradictions between intentional situations and emotional needs) are obtained comparing the two preferred and two rejected forms to the two preferred and two rejected colors. **Basic color tables:** consist in one table of six pairs of squares of basic colors and four other tables (one for each basic color) of six pairs of squares of different shades of each basic color. Subjects are asked to choose their preference of one colored square for each pair in all tables. The four tables of shades of colors allow us to select implicitly basic preferred colors. A regular choice occurs when for every six pairs of choices in the first table the subject chooses one color once, another twice, and another three times. In the opposite cases we have an irregular choice. Psychological states (i.e., frustration, compensation, ambivalence and conflict) are also found on the basis of color choices in all five tables.

### Aims of the Study

The main aim of the study was to assess the emotional state of cognitively impaired patients through the color choice preference in a group of AD patients and compare it with a group of MCI patients and a control group.

Moreover, a secondary aim was to support Lüscher color test as a screening tool to approach emotional problems in cognitively impaired patients.

### Statistical Analysis

A comparison of demographical and clinical features between the three groups was performed using one-way ANOVA followed by Bonferroni *post hoc* test. All dichotomous variables were compared using Pearson’s χ^2^ test. Frequency counts and percentages were calculated separately in each group for all Lüscher color test parameters.

Furthermore, to assess the association between these Lüscher color test parameters and the severity of the disease, measured with the MMSE, Spearman’s Rho correlations were performed. Statistical significance was set at *p* < 0.05 and all analyses were performed using IBM SPSS v.22.

## Results

Seventy-one AD, 50 MCI and 68 controls, matched for age, sex, and education were enrolled. Mean age, sex distribution, education, and general cognitive level (MMSE) of each group are listed in Table 2. Disease duration was respectively of 3.87 ± 1.23 years in the AD group and 2.68 ± 1.12 years in the MCI.

**TABLE 2:** Demographic and clinical data of patients and controls.

	**AD *n* = 71**	**MCI *n* = 50**	**Controls *n* = 68**	***p*/Chi square**
Age (years)	76.08 ± 7.08	77.28 ± 6.3	72.5 ± 8.41	0.013
Sex (male/female)	27/44	24/26	25/43	0.419
Education (years)	8.18 ± 4.4	8.52 ± 4.15	9.46 ± 3.59	0.168
MMSE	17.47 ± 4.9	26.3 ± 2.47	29.31 ± 0.92	<0.001^∗^

*^∗^Statistically significant as *p* < 0.012 (corrected for multiple comparison).*

### Lüscher Color Test Analysis

**Gray table:** state anxiety was detected, respectively in 74.6% of AD patients, 84% of MCI patients and in 89.7% of controls. State anxiety positively correlated with the MMSE score (*r* = 0.207; *p* < 0.05), confirming that a higher score in the MMSE (i.e., control group) corresponds to higher presence of anxiety. **Eight color table:** the order of preference of the first choice of the eight basic and auxiliary colors is reported in Table 3. Cognitively impaired patients, especially AD ones, chose mainly auxiliary colors (violet and brown), while basic colors, especially blue, were chosen by controls. Auxiliary colors choice negatively correlated with the MMSE score (*r* = −0.139; *p* < 0.05), confirming that with a higher severity of disease (i.e., low MMSE score) auxiliary colors are the most chosen. The most rejected color was black in controls (41.2%) and MCI patients (40%) while the most rejected colors in AD patients were black (26.8%) and gray (21.1%). The rejection of basic colors as an indicator of trait anxiety was prevalent among MCI patients (78%) compared to AD patients (67.6%) and controls (67.6%). Green was the prevalent combined color in AD patients (35.5%) while blue was prevalent in MCI (38%) and controls (36%). No differences were found in each group between males and females in choosing preferred colors. **Form table:** the order of preference of the first choice of the seven forms and the prevalence of behavioral masks are reported in Table 4. Patients and controls predominantly chose forms corresponding to auxiliary colors, mainly brown and violet. Moreover, as auxiliary form in particular, AD also chose form #0, corresponding to gray. Behavioral masks were prevalent in the control group (27.9%) compared to AD (18%) and MCI (14.5%) patients. **Basic color tables:** the prevalence of irregular choices and psychological states in all groups are reported in Table 5. Irregular choices were mainly made by cognitively impaired patients, specifically AD (27.5%) and MCI (22%), compared to controls (11.8%); negative correlations were found between irregular choices and the MMSE score (*r* = −0.14; *p* < 0.05). Frustration and ambivalence were prevalent in controls (60.3 and 50%) compared to AD (54.9 and 29.6%) and MCI (46 and 38%) patients; significant differences between groups were found in the ambivalence (*p* < 0.05). Furthermore, positive correlations between the MMSE score and ambivalence were also found (*r* = 0.181; *p* < 0.05), confirming that a higher score in the MMSE (i.e., control group) corresponds to higher presence of ambivalence.

**TABLE 3:** Eight colors first choice preference.

		**AD (%)**	**MCI (%)**	**Controls (%)**
Basic color	1. Blue	11.3	8	20.6
	2. Green	4.2	12	5.9
	3. Red	15.5	14	19.1
	4. Yellow	16.9	18	16.2
Auxiliary color	5. Violet	43.7	42	36.8
	6. Brown	4.2	4	0
	7. Black	1.4	2	1.5
	0. Gray	1.4	0	0
Global preference	Basic color	47.9	52	61.8
	Auxiliary color^∗^	50.7	48	38.2

*^∗^Auxiliary color choice negatively correlates with the MMSE score (*p* < 0.05).*

**TABLE 4:** Forms first choice preferences and behavioral masks.

		**AD (%)**	**MCI (%)**	**Controls (%)**
Basic form	1 (Blue)	2.8	6	1.5
	2 (Green)	15.5	22	22.1
	3 (Red)	8.5	2	1.5
	4 (Yellow)	5.6	16	8.8
Auxiliary form	5 (Violet)	15	24	25
	6 (Brown)	33.8	24	36.8
	0 (Gray)	16.9	6	4.4
Global preference	Basic form	32.4	46	33.9
	Auxiliary form	65.7	54	66.2
	Behavioral masks	18	14.5	27.9

**TABLE 5:** Basic colors choices and psychological states.

	**AD (%)**	**MCI (%)**	**Controls (%)**
Irregular choice^∗^	27.5	22	11.8
Frustration	54.9	46	60.3
Compensation	45.1	42	44.1
Ambivalence^#^	29.6	38	50
Conflict	46.5	50	50

*^∗^Irregular choice negatively correlates with the MMSE score (*p* < 0.05). ^#^Ambivalence positively correlates with the MMSE score (*p* < 0.05).*

## Discussion

Our results suggest that the emotional state of AD patients is generally different compared to normal subjects and that Lüscher color test could be a useful tool to evaluate it due to its simple and rapid administration. Moreover, as we have found in other simple projective tests (the tree-drawing test and the human figure-drawing test) assessing personality and affectivity in AD patients, our sample data of Lüscher color test show a sort of progressive coarctation of the cognitive and emotional life of these patients along the course of the disease (Stanzani Maserati et al., 2015, 2018). In fact, AD patients live with a feeling of personal change due to instability and emotional insecurity, experiencing physical discomfort and a bodily need of being welcomed in a favorable environment. They need calmness, serenity and absence of conflicts and tensions and aspire to a sensitive understanding by someone with whom they can be identified. This emotional state corresponds mainly to violet as prevalent first choice on the eight color table, to gray and black as rejected colors and to brown and gray as prevalent choice on the form table. The worse the degree of cognitive impairment, the greater the intensification of these needs and feelings. Moreover, the greater the severity of cognitive decay, the lower the psychic complexity (less behavioral masks and less occurrence of psychological states like frustration and ambivalence).

The analysis of color choice preferences in our sample provides a sort of “inside view” of patients suffering from AD, which is an experience of physical fragility and insecurity characterized by a significant need for a favorable environment and care (Kojima et al., 2017).

This subjective perspective is therefore different from a typical analysis of behavioral disorders in such type of dementia. In fact the use of projective techniques through the unconscious selection of external stimuli allows us to infer the internal experience of AD patients because they perform these types of tests differently from normal subjects since personality and affectivity, as background, include their cognitive functional aspects.

Differently from AD, MCI patients are more anxious even if they feel less fragile. Thus anxiety could probably be an expression of an aspecific warning signal relative to the perception of their cognitive change. Anxiety in fact is a typical behavioral symptom of this clinical condition whose predictive role in cognitive decline is still debated (Gallagher et al., 2017).

Regarding possible limitations of our study, we focused on anosognosia/metacognitive deficits, i.e., the inability to express a reliable judgment with respect to one’s own state. In fact, it is known that AD patients, due to the progressive neurodegenerative disgregation of cognitive functions, are negatively influenced not only in the main cognitive domains like memory, executive, praxic, and language functions, but also in the self-awareness of their performance (Mograbi et al., 2009). Therefore, in AD patients an output bias could exist in performing the emotional color preference choice. In this regard, previous studies highlighted that, although self-awareness dysfunctions are described and could be part of the global AD pathology i.e., executive and/or mnemonic anosognosia, AD patients’ emotional reactions are to be considered not biased by the neurodegenerative disorder despite other self-conscious deficits (Agnew and Morris, 1998; Mograbi et al., 2009; Shaked et al., 2014). In conclusion, patients with AD show on the emotional/pre-verbal side, a normal implicit awareness (Martyr et al., 2011; Mograbi et al., 2012; Mograbi and Morris, 2013). It is thus conceivable that Lüscher color test, by using colors and basing on a pre-verbal task, could be used as a tool suitable to explore the emotional state in cognitively impaired patients.

However, the validity and reliability of Lüscher color test is still debated (Kertzman et al., 2003). Some authors advocated the validity of the test (French and Alexander, 1972; Carmer et al., 1974; Rahn, 1976; Adels, 1978; Ledford and Hoke, 1981) or suggested a weak validity (Donnelly, 1974; Donnelly, 1977), while others showed no validity (Braün and Bonta, 1979; Stimpson and Stimpson, 1979; Corotto and Hafner, 1980; Holmes et al., 1984, 1986; Picco and Dzindolet, 1994). In particular, Holmes supposed a Burnum effect in the interpretation, which is the reason why the test’s interpretative statements may appear valid, i.e., because they are so general that they could describe almost anyone (Holmes et al., 1986). Authors also showed that there is no relationship between Lüscher color test and the Minnesota Multiphasic Personality Inventory (MMPI) (Holmes et al., 1984; Kertzman et al., 2003). Donnelly, however, taking into account literature and personal data about the correlation between Lüscher color test and scores on the Taylor-Johnson Temperament Analysis (Donnelly, 1977), concluded that “Lüscher test is probably not valid in terms of specific personality descriptions” but that it “might be a useful tool in spite of the specific criticisms voiced” and that “the test may have usefulness as a quick indicator of emotional problems that are extensive enough to warrant further investigation” and this was the critical point of view to which we have referred in our work.

Regarding the use of the test in clinical pathology, previous studies evaluated the use of the color choice test in aging, in neurodegenerative diseases and in other medical conditions (Strenski et al., 1970; Cohen and Hunter, 1978; Corotto and Hafner, 1980; De Leo and Magni, 1982; Holmes et al., 1985a, b; Kuloglu et al., 2002; Volpato, 2008; Zilio, 2013; Savio and Zanardo, 2015; Zanardo et al., 2017).

The psychiatric condition has been explored showing conflicting data since patient groups are often poorly described, not comparable, and relationships are weak (Strenski et al., 1970; Cohen and Hunter, 1978; Corotto and Hafner, 1980; De Leo and Magni, 1982; Holmes et al., 1985a, b; Kuloglu et al., 2002). In general, yellow (Holmes et al., 1985a) and red (Corotto and Hafner, 1980; Holmes et al., 1985a) have been described as preferred colors in groups of generic hospitalized psychiatric patients while green (Kuloglu et al., 2002) has been chosen first by both anxious or depressed patients and by psychotics that are also reported to prefer black (Strenski et al., 1970). Data concerning a supposed general preference for violet by psychiatric patients has not been confirmed (Holmes et al., 1985b) nor has the rejection of yellow by depressed patients (Cohen and Hunter, 1978; De Leo and Magni, 1982).

In a group of old aged normal cognitive subjects living in a retirement home, data from Lüscher color test showed a prevalent rejection of yellow as an expression of becoming withdrawn and gray as a need of protection (Zilio, 2013), while a group of parkinsonian patients expressed a preference for green in the attempt to control their condition and a rejection of blue and red as indicators of depression (Volpato, 2008). In our AD patients the feeling of fragility and the need of a sensitive understanding were prevalent thus expressing different existential illness conditions if compared to the group of parkinsonian patients. Probably different pathological conditions could specifically influence different color choice preferences.

Sex differences in color preferences have been also explored showing heterogeneous data (Braün and Bonta, 1979; Seefeldt, 1979; Stimpson and Stimpson, 1979; Hafner and Corotto, 1980; Silver and Mc Culley, 1988; Silver and Ferrante, 1995; Wijk et al., 1999a, b, 2002). In general, men choose blue and red more frequently while women choose yellow, black and violet (Silver and Mc Culley, 1988; Silver and Ferrante, 1995). The prevalent choice of yellow by females is frequently reported (Braün and Bonta, 1979; Seefeldt, 1979; Silver and Mc Culley, 1988) even if a prevalent choice of the same color by males compared to females has been described (Stimpson and Stimpson, 1979). Anyway, sex seems to be independent of color preference (Hafner and Corotto, 1980) and the rank order of color preferences is stable with age in males and females (Silver and Ferrante, 1995; Wijk et al., 1999b) as well as in AD patients (Wijk et al., 1999a, 2002). In our AD patients, no differences were found between males and females also in choosing preferred colors.

Finally, AD patients have more color vision deficiencies than healthy subjects (Pache et al., 2003), therefore it could be speculated that there is also a significant physiological influence on color choice. Although our study is not designed to clarify this issue, it should be taken into account that color vision deficiencies in AD are not specific (Pache et al., 2003) and that, when present, they are not as severe as in other types of neurodegenerative dementias like that with Lewy bodies (Flanigan et al., 2018). It is also known that color discrimination ability in AD patients is significantly better in the yellow and red area but it is also evident that the severity of dementia did not affect the ability to rank colors in order of preference (Wijk et al., 1999a) and that the preference order for colors remains relatively stable also in normal aging (Wijk et al., 1999b) since color perception is qualitative and well preserved throughout life despite older age and AD (Wijk et al., 1999b, 2002).

There are some limitations in this study. First, Lüscher color test is more suitable to explore emotional state and non-stable traits of personality. Another limitation of the present study is that no correlation has been performed between Lüscher color test scores and standardized anxiety and depression scales. Third, although all patients underwent a general cognitive assessment with the MMSE, a fully comprehensive neuropsychological battery had not been administered. Moreover, further follow-up studies are needed to better study changes in the color choice preference and physiological influences over time along with the progression of the disease and to explore the neuroanatomical association of the color choice preferences, using also magnetic resonance techniques, so as to confirm these data in larger and more homogeneous groups of cognitively impaired patients of different types.

## Conclusion

Knowledge of the inner emotional life of Alzheimer’s patients is mandatory to understand the subjective motivation and perspective of their behavior. However, AD cognitive deterioration does not often allow patients to adequately communicate their needs and existential perspectives, thus they are often not understood, which results in the development of behavioral disorders.

Therefore, the awareness of clinicians about the existential fragility and insecurity of such type of patients allows us to better manage their behavioral disturbances, their quality of life and that of their caregivers. Moreover, this strongly emphasizes the importance of a reliable and gentle care of demented patients and the social necessity of educated and empathic caregivers.

Lüscher color test, like other projective techniques, such as the tree-drawing test and the human figure-drawing test, allows us to approach the evaluation of these patients in a simpler and unconventional way and it could be considered as an appropriate screening tool to approach their emotional problems.

## Data Availability

All datasets generated for this study are included in the manuscript and/or the supplementary files.

## Ethics Statement

All subjects gave consent to personal data processing for research purposes and the protocol was approved by the Local Ethical Committee (v. 2.0 September 2017). All subjects gave their informed consent to the study according to the Declaration of Helsinki.

## Author Contributions

MSM: ideation, methodology, and drafting of the article. MM: statistical analysis and processing. FM: data collection and processing. RD’O, FO, RP, and MDM: data collection. CT: methodology and supervision. RLo and RLi: methodology and supervision. SC: methodology, supervision, and tutoring.

## Conflict of Interest Statement

The authors declare that the research was conducted in the absence of any commercial or financial relationships that could be construed as a potential conflict of interest.

## References

[B1] Adels J. (1978). An assessment of Luscher’s eight color test as a screening instrument for emotional disturbance in school children. *Diss. Abstr. Int.* 39 967–968.

[B2] Agnew S. K. Morris R. G. (1998). The heterogeneity of anosognosia for memory impairment in Alzheimer’s disease: a review of the literature and a proposed model. *Aging Ment. Health* 2 9–15. 16324727

[B3] Albert M. S. DeKosky S. T. Dickson D. Dubois B. Feldman H. H. Fox N. C. (2011). The diagnosis of mild cognitive impairment due to Alzheimer’s disease: recommendations from the national institute on aging-Alzheimer’s association workgroups on diagnostic guidelines for Alzheimer’s disease. *Alzheimers Dement.* 7 270–279. 10.1016/j.jalz.2011.03.008 21514249 PMC3312027

[B4] American Psychiatric Association, (2013). *Diagnostic and Statistical Manual of Mental Disorders*, 5th Edn Arlington, VA: American Psychiatric Association.

[B5] Braün C. M. Bonta J. L. (1979). Cross-cultural validity, reliability, and stimulus characteristics of the Lüscher color test. *J. Pers. Assess.* 43 459–460. 10.1207/s15327752jpa4305_3 512812

[B6] Carmer J. C. Craddick R. A. Smith E. W. (1974). An investigation of the luscher color test personality descriptions. *Int. J. Symbol.* 5 1–6.

[B7] Clare L. Nelis S. M. Martyr A. Roberts J. Whitaker C. J. Markova I. S. (2012). The influence of psychological, social and contextual factors on the expression and measurement of awareness in early-stage dementia: testing a biopsychosocial model. *Int. J. Geriatr. Psychiatry* 27 167–177. 10.1002/gps.2705 21425345

[B8] Cohen E. Hunter I. (1978). Severity of depression differentiated by a color selection test. *Am. J. Psychiatry* 135 611–612. 10.1176/ajp.135.5.611 645963

[B9] Corotto L. V. Hafner J. L. (1980). The Lüscher color test: relationship between color preferences and behavior. *Percept. Mot. Skills* 50:1066. 10.2466/pms.1980.50.3c.1066 7413380

[B10] De Leo D. Magni G. (1982). Differentiating depression severity by color selection: a failure to replicate. *Am. J. Psychiatry* 139:846. 10.1176/ajp.139.6.846a 7081505

[B11] Dermody N. Wong S. Ahmed R. Piguet O. Hodges J. R. Irish M. (2016). Uncovering the neural bases of cognitive and affective empathy deficits in Alzheimer’s Disease and the behavioral-variant of frontotemporal dementia. *J. Alzheimers Dis.* 53 801–816. 10.3233/JAD-160175 27258418

[B12] Donnelly F. A. (1974). The Luscher color test: reliability and selection preferences by college students. *Psychol. Rep.* 34 635–638. 10.2466/pr0.1974.34.2.635 4595043

[B13] Donnelly F. A. (1977). The Luscher color test: a validity study. *Percept. Mot. Skills* 44 17–18. 10.2466/pms.1977.44.1.17 7312539

[B14] Fernandez-Duque D. Hodges S. D. Baird J. A. Black S. E. (2010). Empathy in frontotemporal dementia and Alzheimer’s disease. *J. Clin. Exp. Neuropsychol.* 32 289–298. 10.1080/13803390903002191 19544133

[B15] Fischer A. Landeira-Fernandez J. Sollero de Campos F. Mograbi D. C. (2019). Empathy in Alzheimer’s disease: review of findings and proposed model. *J. Alzheimers Dis.* 69 921–933. 10.3233/JAD-180730 [Epub ahead of print]. 31104016

[B16] Flanigan P. M. Khosravi M. A. Leverenz J. B. Tousi B. (2018). Color vision impairment differentiates Alzheimer dementia from dementia with lewy bodies. *J. Geriatr. Psychiatry Neurol.* 31 97–102. 10.1177/0891988718767579 29658429

[B17] Folstein M. F. Folstein S. E. McHugh P. R. (1975). Mini-mental state. A practical method for grading the cognitive state of patients for the clinician. *J. Psychiatr. Res.* 12 189–198. 1202204 10.1016/0022-3956(75)90026-6

[B18] French C. Alexander B. (1972). The Luscher color test: an investigation of validity and underlying assumptions. *J. Pers. Assess.* 36 361–365. 10.1080/00223891.1972.10119772

[B19] Gallagher D. Fischer C. E. Iaboni A. (2017). Neuropsychiatric symptoms in mild cognitive impairment. *Can. J. Psychiatry* 62 161–169. 10.1177/0706743716648296 28212495 PMC5317015

[B20] Gallassi R. Oppi F. Poda R. Scortichini S. Stanzani Maserati M. Marano G. (2010). Are subjective cognitive complaints a risk factor for dementia? *Neurol. Sci.* 31 327–336. 10.1007/s10072-010-0224-6 20182898

[B21] Hafner J. L. Corotto L. V. (1980). Age, sex, race, and the Lüscher color test. *Percept. Mot. Skills* 50 1144–1146. 10.2466/pms.1980.50.3c.1144 7413388

[B22] Holmes C. B. Buchannan J. A. Dungan D. S. Reed T. (1986). The Barnum effect in luscher color test interpretation. *J. Clin. Psychol.* 42 133–136. 10.1002/1097-4679(198601)42:1<133::aid-jclp2270420122>3.0.co;2-7

[B23] Holmes C. B. Fouty H. E. Wurtz P. J. Burdick B. M. (1985a). The relationship between color preference and psychiatric disorders. *J. Clin. Psychol.* 41 746–749. 10.1002/1097-4679(198511)41:6<746::aid-jclp2270410603>3.0.co;2-q 4077997

[B24] Holmes C. B. Fouty H. E. Wurtz P. J. (1985b). Choice of Luscher’s color violet in a psychiatric sample. *Percept. Mot. Skills* 60:402. 10.2466/pms.1985.60.2.402 4000853

[B25] Holmes C. B. Wurtz P. J. Waln R. F. Dungan D. S. Joseph C. A. (1984). Relationship between the Luscher color test and the MMPI. *J. Clin. Psychol.* 40 126–128. 10.1002/1097-4679(198401)40:1<126::aid-jclp2270400123>3.0.co;2-a 6746918

[B26] Kertzman S. Spivak B. Ben-Nahum Z. Vainder M. Reznik I. Weizman A. (2003). Variability of color choice in the Lüscher color test-sex differences. *Percept. Mot. Skills* 97 647–656. 10.2466/pms.2003.97.2.647 14620256

[B27] Kojima G. Liljas A. Iliffe S. Walters K. (2017). Prevalence of frailty in mild to moderate Alzheimer’s disease: a systematic review and meta-analysis. *Curr. Alzheimer Res.* 14 1256–1263. 10.2174/1567205014666170417104236 28413984

[B28] Kuloglu M. Atmaca M. Tezcan A. E. Unal A. Gecici O. (2002). Color and number preferences of patients with psychiatric disorders in eastern Turkey. *Percept. Mot. Skills* 94 207–213. 10.2466/pms.2002.94.1.207 11883563

[B29] Lane C. A. Hardy J. Schott J. M. (2018). Alzheimer’s disease. *Eur. J. Neurol.* 25 59–70. 10.1111/ene.13439 28872215

[B30] Ledford R. S. Hoke W. E. (1981). Self-report as a validity check for the Lüscher color test. *Percept. Mot. Skills* 53 545–546. 10.2466/pms.1981.53.2.545 7312539

[B31] Lüscher M. (1948). *Psychologie Der Farben.* Basel: Test-Verlag.

[B32] Lüscher M. Scott I. (1969). *The Lüscher Colour Test.* New York, NY: Random House.

[B33] Magni E. Binetti G. Bianchetti A. Rozzini R. Trabucchi M. (1996). Mini-mental state examination: a normative study in Italian elderly population. *Eur. J. Neurol.* 3 1–5. 10.1080/21622965.2018.1522590 21284770

[B34] Martyr A. Clare L. Nelis S. M. Roberts J. L. Robinson J. U. Roth I. (2011). Dissociation between implicit and explicit manifestations of awareness in early stage dementia: evidence from the emotional stroop effect for dementia-related words. *Int. J. Geriatr. Psychiatry* 26 92–99. 10.1002/gps.2495 21157854

[B35] McKhann G. M. Knopman D. S. Chertkow H. Hyman B. T. Jack C. R. Jr. Kawas C. H. (2011). The diagnosis of dementia due to Alzheimer’s disease: recommendations from the National Institute on aging-Alzheimer’s association workgroups on diagnostic guidelines for Alzheimer’s disease. *Alzheimers Dement.* 7 263–269. 10.1016/j.jalz.2011.03.005 21514250 PMC3312024

[B36] Mograbi D. C. Brown R. Salas C. E. Morris R. G. (2012). Emotional reactivity and awareness of task performance in Alzheimer’s disease. *Neuropsychologia* 50 2075–2084. 10.1016/j.neuropsychologia.2012.05.008 22609573

[B37] Mograbi D. C. Brown R. G. Morris R. G. (2009). Anosognosia in Alzheimer’s disease – The petrified self. *Conscious. Cogn.* 18 989–1003. 10.1016/j.concog.2009.07.005 19683461

[B38] Mograbi D. C. Morris R. G. (2013). Implicit awareness in anosognosia: Clinical observations, experimental evidence, and theoretical implications. *Cogn. Neurosci.* 4 181–197. 10.1080/17588928.2013.833899 24251606

[B39] Pache M. Smeets C. H. Gasio P. F. Savaskan E. Flammer J. Wirz-Justice A. (2003). Colour vision deficiencies in Alzheimer’s disease. *Age Ageing* 32 422–426. 12851187 10.1093/ageing/32.4.422

[B40] Petersen R. C. Roberts R. O. Knopman D. S. Boeve B. F. Geda Y. E. Ivnik R. J. Jr. . (2009). Mild cognitive impairment: ten years later. *Arch. Neurol.* 66 1447–1455. 10.1001/archneurol.2009.266 20008648 PMC3081688

[B41] Picco R. D. Dzindolet M. T. (1994). Examining the Lüscher color test. *Percept. Mot. Skills* 79 1555–1558. 10.2466/pms.1994.79.3f.1555 7870544

[B42] Rahn R. (1976). Lüscher color theory: civilians and criminals. *Art Psychothe.* 3 145–155. 10.1016/0090-9092(76)90020-x

[B43] Savio F. Zanardo V. (2015). Unconscious dynamics in twin pregnancy emerging from the lüscher color test. *J. Matern. Fetal Neonatal Med.* 28 199–203. 10.3109/14767058.2014.907263 24660898

[B44] Seefeldt F. M. (1979). The Luscher color test: sex differences in color preference. *Percept. Mot. Skills* 48 896–898. 10.2466/pms.1979.48.3.896 482042

[B45] Shaked D. Farrel M. Huey E. Metcalfe J. Cines S. Karlawish J. (2014). Cognitive correlates of metamemory in Alzheimer’s disease. *Neuropsychology* 28 695–705. 10.1037/neu0000078 24819066 PMC4143443

[B46] Silver N. C. Ferrante R. (1995). Sex differences in color preferences among an elderly sample. *Percept. Mot. Skills* 80 920–922. 10.2466/pms.1995.80.3.920 7567412

[B47] Silver N. C. Mc Culley W. L. (1988). Sex and racial differences in color and number preferences. *Percept. Mot. Skills* 66 295–299. 10.2466/pms.1988.66.1.295

[B48] Stanzani Maserati M. D’Onofrio R. Matacena C. Sambati L. Oppi F. Poda R. (2018). Human figure drawing distinguishes Alzheimer’s patients: a cognitive screening test study. *Neurol. Sci.* 39 851–855. 10.1007/s10072-018-3288-3 29455399

[B49] Stanzani Maserati M. Matacena C. Sambati L. Oppi F. Poda R. De Matteis M. (2015). The tree-drawing test (Koch’s Baum test): a useful aid to diagnose cognitive impairment. *Behav. Neurol.* 2015:534681. 10.1155/2015/534681 26175548 PMC4484840

[B50] Stimpson D. V. Stimpson M. F. (1979). Relation of personality characteristics and color preferences. *Percept. Mot. Skills* 49 60–62. 10.2466/pms.1979.49.1.60 503760

[B51] Strenski A. Poyson J. M. Muzecari L. H. (1970). Preferences and dislikes for color among chronic schizophrenics. *J. Clin. Psychol.* 26 310–311. 10.1002/1097-4679(197007)26:3<310::aid-jclp2270260317>3.0.co;2-6

[B52] Volpato C. (2008). *Il Test Dei Colori Di Lüscher Nella Malattia Di Parkinson: Un Tentativo Di Convergenza Tra Teorie Psicodinamiche Della Personalità e Neuroscienze. Cisspat, Padova in Del Longo N.. Il Test Dei Colori Di Lüscher. Manuale Di Diagnostica Per L’età Adulta.* Milan: Franco Angeli.

[B53] Wijk H. Berg S. Bergman B. Hanson A. B. Sivik L. Steen B. (2002). Colour perception among the very elderly related to visual and cognitive function. *Scand. J. Caring Sci.* 16 91–102. 10.1046/j.1471-6712.2002.00063.x 11985755

[B54] Wijk H. Berg S. Sivik L. Steen B. (1999a). Colour discrimination, colour naming and colour preferences among individuals with Alzheimer’s disease. *Int. J. Geriatr. Psychiatry* 14 1000–1005. 10.1002/(sici)1099-1166(199912)14:12<1000::aid-gps46>3.3.co;2-5 10607966

[B55] Wijk H. Berg S. Sivik L. Steen B. (1999b). Color discrimination, color naming and color preferences in 80-year olds. *Aging Clin. Exp. Res.* 11 176–185. 10.1007/bf03399660 10476313

[B56] Zanardo V. Gabrieli C. Volpe F. Savio F. Straface G. Soldera G. (2017). Postpartum unconscious dynamics emerging from the lüscher color test in ethiopian women. *J. Matern. Fetal Neonatal Med.* 30 1446–1449. 10.1080/14767058.2016.1219985 27485703

[B57] Zilio M. (2013). *Il Test di Lüscher in Terza Età. Cisspat, Padova in Del Longo N. Il Test Dei Colori Di Lüscher. Manuale Di Diagnostica Per L’età Adulta.* Milano: Franco Angeli.

